# Diverse and inclusive human resource practices and employee creativity: the psychological fit mechanisms and the role of inclusive leadership

**DOI:** 10.3389/fpsyg.2025.1679943

**Published:** 2025-11-27

**Authors:** Zhixing Zhao, Jiajia Ren

**Affiliations:** 1 School of Economics and Management, Sichuan Normal University, Chengdu, China; 2 Xuzhou Central Hospital, Xuzhou, China

**Keywords:** diverse and inclusive human resource practices, supplementary fit, complementary fit, employee creativity, inclusive leadership

## Abstract

**Introduction:**

In the context of a VUCA environment and an increasingly diverse workforce, stimulating employee creativity has become a key driver of sustained organizational innovation. Diverse and Inclusive Human Resource Practices (DIHRP) are a series of systematic management activities that enhance employees’ diversity and inclusion capabilities, stimulate their diversity and inclusion motivation, and provide corresponding opportunities. However, the potential mechanisms and boundary conditions of DIHRP’s impact on employee creativity remain underexplored.

**Methods:**

This study draws on social identity theory and conservation of resources theory to construct a moderated mediation model. Based on survey data from 372 employees, hierarchical regression and bootstrap analysis were used to test the model.

**Results:**

The results show that: (1) DIHRP significantly promotes employee creativity; (2) both supplementary fit and complementary fit mediate the relationship between DIHRP and creativity; and (3) inclusive leadership positively moderates the direct and indirect effects of DIHRP, thereby strengthening the association between DIHRP and creativity.

**Discussion:**

This study reveals the psychological mechanism linking human resource practices to creative outcomes, enriches the theoretical perspectives of DIHRP and leadership style, and provides practical implications for optimizing talent management in different organizational environments.

## 1 Introduction

As the external environment continues to intensify with VUCA (volatility, uncertainty, complexity, and ambiguity), companies must continuously innovate to survive (Jia et al., 2024). *Employee creativity* is a core driver for organizations to achieve sustainable competitive advantage in a complex and volatile business environment (Zhang et al., 2023). However, as the workforce becomes increasingly diverse in terms of gender, age, and cultural background, while leveraging the potential of diversity, organizations also face the universal challenge of effectively managing and stimulating the creativity of all members (Ahmed et al., 2024). The traditional control-oriented human resource practice paradigm has become increasingly limited in this context, while diverse and inclusive human resource practices that emphasize both diversity and inclusion are demonstrating their unique value (Zhao et al., 2020).

*Diverse and Inclusive Human Resource Practices (DIHRP)* are a series of systematic management activities that enhance employees’ diversity and inclusion capabilities, stimulate their diversity and inclusion motivation, and provide corresponding opportunities (Zhao et al., 2020). Therefore, DIHRP demonstrates significant potential for enhancing employee capabilities, stimulating work motivation, and expanding development opportunities, particularly in environments with a highly diverse workforce (Zhao et al., 2022). Initial academic research on diversity and inclusion management has focused on informal support mechanisms. For example, in the era of economic globalization, how organizations manage cross-border, cross-cultural, and intergenerational workforce mobility through informal institutions (inclusive culture and climate, as well as leadership) (Hu et al., 2023; Roberson and Perry, 2022). Furthermore, while multidimensional differences in gender, age, educational background, ethnicity, religious beliefs, and family structure offer rich cognitive resources and enhanced potential for innovative collaboration, they also place higher demands on organizational management systems (Gümüsay et al., 2020). Although scholars have begun to pay attention to formal DIHRP in recent years and have preliminarily confirmed its positive effects on employee well-being (Moore et al., 2017), thriving at work (Zhao et al., 2022), and individual innovative behavior (Zhao et al., 2020), research on how DIHRP affects employee creativity is still in its infancy, and its internal psychological mechanisms and boundary conditions are still unclear.

To address these issues, we draw on Social Identity Theory (SIT) and Conservation of Resources (COR) theory to construct a moderated dual-path model. We hypothesize that, drawing on SIT (Brown, 2000), DIHRP can enhance employee creativity by fostering a sense of belonging and shared values, fostering employee *supplementary fit* (the degree of similarity between individuals and organizations in values; Muchinsky and Monahan, 1987). Furthermore, drawing on COR theory (Hobfoll, 1989), DIHRP can provide employees with diverse development resources, a supportive performance evaluation system, etc., to enhance employee *complementary fit* (the extent to which employees and organizations meet each other’s needs through resource complementarity, Muchinsky and Monahan, 1987), thereby enhancing employee creativity.

Given our interest in understanding when DIHRP enhances employee creativity through ambidextrous fit, we sought to explore the contingency factors that may influence the impact of DIHRP on ambidextrous fit and employee creativity. Previous research suggests that the interaction between formal institutional arrangements (such as HR practices) and informal relationship dynamics (such as leadership style) has a more pronounced impact on employee behavior (Hu et al., 2025). Furthermore, studies have shown that shared leadership and charismatic leadership can moderate the impact of DIHRP on employee creativity (Zhao et al., 2020; Zhao et al., 2022). While these studies have explored the boundary conditions under which leadership style influences DIHRP, a deeper exploration of these boundary conditions remains lacking. *Inclusive leadership*, which refers to leaders respecting and embracing diversity, supporting autonomy, and encouraging voice in their management of subordinates (Roberson and Perry, 2022), may enhance employees’ cognition and internalization of DIHRP, thereby enhancing its actual effectiveness. Therefore, exploring how DIHRP (hard rules) and inclusive leadership (soft support) work together to shape employees’ ambidextrous fit and creativity is crucial to unlocking the “black box” mechanism of DIHRP.

In summary, this study aims to explore the following three specific questions: (1) Does DIHRP effectively predict employee creativity? (2) Do supplementary fit and complementary fit mediate the relationship between DIHRP and employee creativity? (3) Does inclusive leadership moderate the direct and indirect effects of DIHRP? By answering these questions, this study aims to theoretically reveal the psychological mechanisms by which DIHRP enhances employee creativity, enriches SIT and COR, and provides practical insights for optimizing talent management systems in diverse organizations.

## 2 Theoretical background and hypotheses

### 2.1 Diverse and inclusive human resource practices and employee creativity

Existing research on inclusive management has predominantly focused on informal forms of organizational support—such as inclusive leadership, inclusive culture, and inclusive climate (Pless and Maak, 2004; Roberson and Perry, 2022; Wray et al., 2022). In contrast, DIHRP, as a formalized institutional arrangement, remains an emergent yet underexplored area of inquiry. DIHRP refers to the systematic integration of inclusivity principles into core HRM functions, including recruitment, development, deployment, and retention. These practices aim to provide structured support in areas such as respect for individual differences, potential development, tolerance for failure, and motivational enhancement, thereby facilitating a synergistic alignment between organizational performance and employee growth (Moore et al., 2017).

From the perspective of Leader–Member Exchange (LMX), inclusion entails respect, mutual understanding, and shared responsibility (Burmeister et al., 2018); from a relational perspective, it emphasizes active engagement and interaction (Rudolph and Zacher, 2021); and from a justice-oriented perspective, it advocates equitable treatment for marginalized groups (Zhao et al., 2023). By institutionalizing these values, DIHRP serves as a key mechanism that bridges organizational objectives with employees’ lived experiences.

Employee creativity—defined as the ability and tendency to generate novel and useful ideas, processes, products, or services within the workplace (Zhou and George, 2001)—is widely recognized as a critical driver of both individual performance and organizational innovation (Farmer et al., 2003). Creativity reflects an activation of employees’ cognitive resources, domain-specific knowledge, and intrinsic motivation in response to complex problems or opportunities.

In today’s VUCA environment, individuals face the dual pressures of escalating demands and continuous resource depletion. According to COR theory, individuals are motivated to acquire, retain, and invest resources to cope with actual or potential threats of loss (Halbesleben and Wheeler, 2015). DIHRP can serve as a source of psychological and instrumental resources that buffer such stressors while simultaneously catalyzing creative behavior (Aust et al., 2020).

Specifically, DIHRP promotes creativity through the following mechanisms:

Inclusive recruitment practices emphasize diversity and recognition of individual backgrounds and capabilities, thereby reinforcing employees’ confidence in the value of their unique resources and enhancing their creative self-efficacy.

Differentiated training and development systems offer multilayered and personalized learning opportunities, enabling employees to build domain-relevant knowledge, skills, and cognitive flexibility. This resource accumulation helps mitigate stress arising from perceived inadequacy and supports creative expression.

Inclusive performance appraisal and reward systems incorporate diverse evaluation criteria and blended incentive strategies, meeting both material and psychological needs. This fosters an equitable and motivating environment conducive to creativity.

Thus, we hypothesize:

*H1*: DIHRP has a significant positive effect on employee creativity.

### 2.2 DIHRP, supplementary fit, and complementary fit

Person-Organization Fit (P-O Fit) is a core construct in organizational behavior, referring to the compatibility between individuals and organizations (Kristof, 1996). Building upon the classic classification framework of person-organization fit (Muchinsky and Monahan, 1987; Kristof, 1996), we focus on two key perceptions of fit: supplementary fit and complementary fit. Supplementary fit occurs when individuals and organizations share fundamental characteristics, particularly in the shared perception of core values, norms, and identity traits (Kristof, 1996). Complementary fit occurs when both parties fulfill each other’s needs, operationalized through two dimensions: need-supply fit (the extent to which the organization meets individual needs) and demands–abilities fit (the extent to which individuals meet organizational requirements) (Cable and DeRue, 2002). Next, we first discuss the positive impact of DIHRP on employees’ supplementary fit.

According to SIT, when employees perceive a high degree of alignment between their personal values and the organizational identity, they develop stronger organizational identification and belonging—that is, enhanced congruence matching (Kristof, 1996). DIHRP creates a work environment that values and integrates diverse identities through systematic institutional arrangements, thereby profoundly shaping employees’ perceptions of supplementary fit with the organization’s core values.

First, at the level of value shaping, the core principles advocated by DIHRP—such as “respecting differences, recognizing value, and encouraging participation”—transmit strong organizational identity signals through formal regulations and informal cultural dissemination. When employees observe that the organization’s values—such as “fairness,” “inclusivity,” and “empowerment”—align with their own ethical beliefs, a deep resonance occurs. This resonance forms the cornerstone of perceived alignment (Muchinsky and Monahan, 1987).

Second, from the perspective of identity mechanisms, the DIHRP creates a psychologically safe space for employees through practices such as “tolerating employee mistakes and leveraging employee potential,” enabling them to express their unique traits without concealment. According to SIT (Ashforth and Mael, 1989), when organizational identity is attractive and individuals can integrate their authentic selves into the organization, employees perceive stronger similarities and shared characteristics between themselves and the organization. This reinforces the perception that “we” belong to the same group, thereby enhancing supplementary fit.

Finally, from the perspective of social categorization effects, the employee collaboration fostered by DIHRP breaks down barriers between departments and identity groups, creating more opportunities for cross-group interaction. Through frequent, positive interactions, employees more readily discover shared goals and a common destiny, thereby diminishing individual differences and strengthening identification with a shared organizational identity (Silva et al., 2022). Therefore, we hypothesize:

*H2a*: DIHRP has a significant positive effect on employees’ perceived supplementary fit.

According to COR, employees are motivated to acquire and protect resources that are valuable to them (Hobfoll, 1989). DIHRP systematically enhances employees’ perceptions of complementary fit at the needs and capabilities levels by providing key resources and building supportive environments.

First, at the need-supply fit level, DIHRP directly and precisely fulfills employees’ higher-order needs for developmental resources, psychological resources, and opportunity resources through abundant training resources, comprehensive recognition and reward systems, and diverse channels for participatory decision-making. According to COR (Hobfoll, 1989), individuals instinctively seek supportive environments that replenish their resource reserves. When organizations consistently and systematically fulfill these critical employee needs through DIHRP, employees significantly enhance their perception of alignment between “what the organization provides” and “what I need” (Cable and DeRue, 2002).

Second, at the demands-abilities fit level, DIHRP effectively enhances employees’ knowledge, skills, and problem-solving abilities through personalized career development plans that unlock employee potential and empower job designs. This enables employees to more confidently tackle workplace challenges and meet organizational role expectations. Such capability growth, driven by organizational investment, directly strengthens employees’ confidence in the fit between “what I possess” and “what the organization requires.”

Finally, DIHRP fosters psychological safety through its practice of “tolerating employee mistakes,” which itself constitutes a valuable resource (Umrani et al., 2023). This reduces the risk of resource loss from failed innovation attempts, making employees more willing and able to invest existing resources (such as capabilities and time) into uncertain creative activities. Consequently, it further solidifies their perception of complementary fit at the psychological level. Therefore, we hypothesize:

*H2b*: DIHRP has a significant positive effect on employees' perceived complementary fit.

### 2.3 Supplementary fit, complementary fit, and employee creativity

A high degree of supplementary fit can effectively stimulate employee creativity through the psychological pathways of social identity and intrinsic motivation. First, the strong organizational identity triggered by supplementary fit is a core mechanism of SIT (Ashforth and Mael, 1989). When employees perceive shared values and identity traits with their organization, they view organizational success as an extension of personal achievement. This process of internalizing organizational goals as personal objectives sparks deep intrinsic motivation, driving employees to exert efforts beyond role requirements for organizational benefit—including dedicating sustained cognitive resources to explore novel and useful ideas (Jia et al., 2024).

Second, supplementary fit fosters shared mental models. When team members share similar values and interpretations of organizational norms, communication flows more smoothly and collaboration becomes more efficient. This shared understanding reduces interpersonal friction and cognitive expenditure stemming from misunderstandings and conflicting goals, freeing employees to channel their mental energy toward complex, deep-thinking creative tasks (Brown, 2000).

Finally, a sense of belonging rooted in supplementary provides employees with psychological safety. In a “like-minded” environment, employees trust that their unique ideas—even unconventional ones—will be understood by colleagues and fairly evaluated by the organization, rather than criticized or rejected. This security is a crucial prerequisite for taking risks and expressing unconventional perspectives, and risk-taking is an indispensable component of the creative process (Kahn, 1990).

Thus, through three mechanisms—stimulating intrinsic motivation, optimizing collaborative efficiency, and providing psychological safety—supplementary fit lays a solid psychological foundation for the emergence of employee creativity. Therefore, we hypothesize:

*H3a*: Supplementary fit has a significant positive effect on employee creativity.

Complementary fit provides the essential energy and conditions for employees’ creativity through resource pathways and motivation pathways. First, a high level of complementary fit means employees perceive that the organization can consistently provide the resources they need (need-supply fit) and that they possess the capabilities to successfully complete tasks (demands-abilities fit). This perception directly creates a state of resource surplus. According to COR (Halbesleben and Wheeler, 2015), individuals with abundant resources not only possess greater capacity to tackle challenges but also exhibit a stronger propensity for resource investment—that is, a willingness to allocate existing resources toward risky yet potentially high-return activities, such as exploring novel work methods or proposing innovative ideas.

Second, complementary fit, particularly need-supply fit, cultivates employees’ sense of gratitude and willingness to reciprocate. Based on social exchange theory, when employees perceive that the organization has substantially met their needs through DIHRP, they develop a sense of obligation to reciprocate. Demonstrating creativity, as an organizational citizenship behavior that extends beyond basic duties, becomes a key way to express gratitude and maintain reciprocal relationships.

Finally, complementary fit effectively reduces employees’ cognitive load and resource guarding mentality. When employees are free from anxiety over resource scarcity or capability deficits, their cognitive systems are liberated from constant vigilance and defensiveness, thereby gaining the cognitive leeway essential for creative thinking (Wightman and Christensen, 2025). Therefore, we hypothesize:

*H3b*: Complementary fit has a significant positive effect on employee creativity.

### 2.4 The mediating role of supplementary fit and complementary fit

Based on the above discussion, we propose a dual-path moderated mediation model. DIHRP, as a comprehensive organizational management system, promotes employee creativity not simply through direct effects but by enhancing two distinct yet complementary perceptions of person-organization fit: supplementary fit and complementary fit.

Integrating H2a and H3a, we propose that employee supplementary fit mediates the relationship between DIHRP and employee creativity. Specifically, by institutionalizing inclusive values, DIHRP provides employees with an organizational environment that is attractive and relatable in terms of values and identity. This environment encourages employees to have a strong sense of identification with the organizational culture, thereby stimulating their intrinsic motivation to engage in creative exploration for the benefit of the organization. Meanwhile, the psychological safety and shared understanding derived from supplementary fit remove social barriers to the proposal and exchange of creative ideas. Therefore, DIHRP first improves employees’ supplementary fit, then strengthens their intrinsic motivation and psychological safety, and ultimately indirectly drives employee creativity. Therefore, we hypothesize that:

*H4a*: Supplementary fit mediates the relationship between DIHRP and employee creativity.

Integrating H2b and H3b, we propose that employee complementary fit mediates the relationship between DIHRP and employee creativity. Specifically, DIHRP systematically constructs a highly supportive, low-threat work environment by providing development resources, empowering employees, and protecting them from penalties for innovation failure. This leads employees to perceive a high degree of need-supply and demand-capability fit, resulting in a state of resource abundance. According to COR theory, this sense of resource surplus frees employees from the anxiety of resource conservation, making them both capable and willing to invest their cognitive resources and energy in creative activities with uncertainty, manifesting as a form of resource investment behavior. Therefore, DIHRP indirectly fosters employee creativity by first enhancing employees’ complementary fit, then enabling their resource investment, and stimulating reward behavior. Therefore, we hypothesize:

*H4b*: Complementary fit mediates the relationship between DIHRP and employee creativity.

### 2.5 The moderating role of inclusive leadership

Human beings are inherently social and relational, and within organizational contexts, the emotional quality of the relationship between leaders and subordinates plays a critical role in shaping the effectiveness of management systems. As a relationship-oriented leadership style, inclusive leadership is defined as leaders’ demonstration of openness to individual differences, encouragement of employee participation, and support for marginalized or underrepresented groups, thereby fostering a team climate characterized by respect, diversity, openness, and empowerment (Orekoya, 2023). Inclusive leaders exhibit openness, accessibility, and availability in their interactions, actively listen to diverse perspectives, support self-expression, respond to emotional cues, and build trust and psychological safety to unlock employee potential (Umrani et al., 2023).

In the implementation of DIHRP, leadership behaviors serve as critical situational moderators that shape employees’ perceptions and interpretations of HR policies. Specifically, when leaders exhibit inclusive behaviors—such as respect for individual differences and supportive responses—across key HR touchpoints, including selection, placement, development, and feedback, employees are more likely to perceive the organization as humane, emotionally responsive, and trustworthy. This enhances their psychological attachment and engagement.

According to COR Theory, emotional affirmation and support from leaders constitute valuable psychological resources that buffer stress and enhance intrinsic motivation. When DIHRP is reinforced by consistent, inclusive leadership behaviors, employees experience a synergistic gain in emotional resources. This, in turn, fosters more positive affective states and facilitates creativity. Particularly in contexts of high uncertainty and job complexity, inclusive leadership helps employees regulate emotions, clarify goals, and activate their creative capacities.

In light of the above, we propose the following hypothesis:

*H5*: Inclusive leadership positively moderates the relationship between DIHRP and employee creativity, such that the effect of DIHRP on employee creativity is stronger when the level of inclusive leadership is high.

Grounded in SIT, individuals strive for a sense of belonging and identity within organizations, particularly when their personal values are perceived to be congruent with organizational goals and cultural orientations (Wang et al., 2024). This perceived value alignment enhances organizational identification, which in turn fosters positive attitudes and constructive behaviors. DIHRP enhances such alignment by systematically recognizing and respecting employee differences, thereby signaling the organization’s genuine commitment to diversity in culture, background, and values. This inclusive institutional stance enables employees from diverse backgrounds to feel acknowledged, understood, and supported, ultimately strengthening their identification with the organization’s cultural and normative system. Such identity-based recognition serves as a core psychological foundation for developing perceptions of supplementary fit.

Meanwhile, inclusive leadership, as a relationship-oriented leadership style, emphasizes interpersonal harmony, emotional support, and individualized care (Umrani et al., 2023). Through behaviors such as active listening, empathetic responsiveness, and emotional recognition, inclusive leaders reinforce employees’ emotional bonds with the organization beyond the formal institutional context. By narrowing the emotional distance between employees and the organization, inclusive leaders enhance employees’ perceived congruence with HR systems and policy intent. In this sense, inclusive leadership functions as a contextual catalyst that amplifies the effect of DIHRP on employees’ perceptions of supplementary fit.

In addition, within the diverse and differentiated environment established by DIHRP, organizations adopt flexible talent selection mechanisms, multiple development pathways, and diversified incentive systems to fulfill employees’ heterogeneous needs for capability development, resource access, and career advancement. These practices lay a strong foundation for complementary fit by facilitating alignment between employee competencies and organizational demands, as well as between organizational support and employee needs. However, in practice, employees may still experience emotional strain, frustration, or even burnout due to role overload, skill inadequacy, or resource misalignment.

In such situations, inclusive leadership, through high levels of emotional intelligence and social support behaviors, can play a critical role in helping employees regulate emotions, clarify expectations, and overcome growth-related obstacles. By doing so, leaders enhance employees’ perceptions of both demands–abilities fit and need–supply fit, thereby reinforcing the impact of DIHRP on complementary fit.

Based on this reasoning, we propose the following hypotheses:

*H6a*: Inclusive leadership moderates the relationship between DIHRP and employees' perceived supplementary fit, such that the relationship is stronger when the level of inclusive leadership is high.

*H6b*: Inclusive leadership moderates the relationship between DIHRP and employees’ perceived complementary fit, such that the relationship is stronger when the level of inclusive leadership is high.

According to COR Theory, employees perceive both material resources (e.g., compensation, development opportunities) and psychological resources (e.g., recognition, respect) provided by the organization as crucial means to buffer stress and facilitate personal growth (Hu et al., 2023). DIHRP fosters a supportive climate by embedding inclusion in the organization’s culture, value orientation, and institutional arrangements. When such practices are implemented under high levels of inclusive leadership, employees are more likely to perceive a strong alignment between their personal values, behavioral orientations, and organizational goals. This enhanced sense of value congruence contributes to a stable and profound perception of supplementary fit, which in turn promotes positive affect, organizational identification, and work enthusiasm—key psychological conditions for the activation of creativity.

Meanwhile, inclusive leaders, who are characterized by high emotional intelligence and relational sensitivity, are adept at recognizing challenges and emotional tensions that employees may experience at work. By offering timely guidance and socio-emotional support, such leaders help employees navigate role-related stress and developmental obstacles. Within the development-oriented environment established by DIHRP, leaders’ emotion-regulation capabilities and encouragement behaviors further promote employees’ perceptions of demands–abilities fit and need–supply fit, the two subdimensions of complementary fit. These perceptions strengthen trust in organizational support and increase employees’ belief in the utility of organizational resources for their own growth and performance.

Thus, we propose the following moderated mediation hypotheses:

*H7a*: Inclusive leadership moderates the indirect effect of DIHRP on employee creativity via supplementary fit, such that the indirect effect is stronger when the level of inclusive leadership is high.

*H7b*: Inclusive leadership moderates the indirect effect of DIHRP on employee creativity via complementary fit, such that the indirect effect is stronger when the level of inclusive leadership is high.

**Figure 1:**
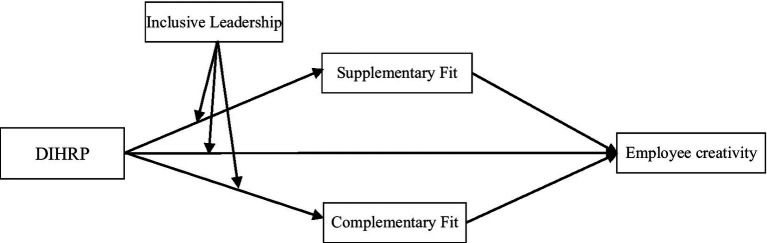
Conceptual model.

In summary, the theoretical concept is shown in Figure 1.

## 3 Method

### 3.1 Sample and procedure

This study surveyed employees from 40 companies located in Sichuan, Guangdong, Hubei, Jiangsu, Shaanxi, and Zhejiang provinces in China, covering a diverse range of industries including information and communication technology, mechanical manufacturing, construction and engineering, financial services, and hospitality and tourism. Data were collected through on-site distribution and return of paper-based questionnaires.

To enhance the temporal separation of variables and minimize common method bias, a three-wave survey design was adopted. Data collection took place between February and May 2025, with a one-month interval between each wave. At Time 1 (February 2025), DIHRP and Inclusive Leadership were assessed. A total of 530 questionnaires were distributed, and 510 were returned. After excluding 24 invalid responses, 486 valid questionnaires were retained. At Time (March 2025), supplementary fit and complementary fit were measured among the valid T1 respondents. Four hundred sixty questionnaires were returned, of which 35 were excluded due to missing data or inconsistencies, yielding 425 valid responses. At Time (April 2025), employee creativity was assessed by tracking the valid T2 participants. Four hundred five responses were received, and after eliminating 33 invalid questionnaires, the final sample comprised 372 valid responses, resulting in an overall effective response rate of 70.19%.

Among the survey respondents, 56.45% were male, and 43.55% were female; 18–25 years 18.01%, 26–35 years 36.83%, 36–55 years 37.37%, 56 years and above 7.8%; 19.35% junior college or below, 27.42% bachelor’s degree, 29.84% master’s degree, 23.39% doctorate or above; 63.17% Frontline employees, 22.85% Middle-level managers, 13.98% Senior managers; 19.35% information and communication technology, 19.09% mechanical manufacturing, 16.13% construction and engineering, 15.05% financial services, 24.20% hospitality and tourism, and 6.18% others.

### 3.2 Measures

The survey was conducted in Chinese; we translated the original items of each measure into Chinese using Brislin’s (1970) forward and backward process. Unless otherwise stated, items were evaluated on a five-point Likert scale that ranged from 1 (strongly disagree) to 5 (strongly agree).

#### 3.2.1 Diverse and inclusive human resource practices

Diverse and inclusive human resource practices was measured with a five-dimensional 20-item scale adapted from Zhao et al. (2022). A sample item is “My company does not discriminate in recruitment and selection based on gender, ethnicity, religion, native place or dialect, etc.” (*α* = 0.918).

#### 3.2.2 Supplementary fit and complementary fit

Both supplementary fit and complementary fit were measured using scales developed by Cable and DeRue (2002). The supplementary fit scale consisted of three items, focusing on value congruence between the employee and the organization. A sample item is: “My personal values match my organization’s values and culture.” (*α* = 0.921). Complementary fit was assessed across two dimensions: need–supply fit and demands–abilities fit, each measured with three items, for a total of six items. Sample items include: “The resources provided by my job meet my expectations for what I need in a job” (need–supply fit), and “The demands of my job match well with my skills and abilities” (demands–abilities fit) (*α* = 0.935).

#### 3.2.3 Employee creativity

Employee creativity was measured with the 4-item scale of Baer and Oldham (2006). Sample items were “I usually have a good source of creativity,” “I often come up with creative solutions to problems at work.” (*α* = 0.934).

#### 3.2.4 Inclusive leadership

Inclusive leadership was measured with the three-dimensional nine items inclusive leadership scale by Carmeli et al. (2010), including openness (e.g., “Leaders are willing to listen to my new views, new ideas and new ideas”), effectiveness (e.g., “On professional issues, leaders can help me”) and accessibility (e.g., “Leaders are willing to listen to my demands”) (*α* = 0.904).

#### 3.2.5 Control variables

This study controlled for a series of individual characteristic variables that may influence employee creativity to more clearly reveal the intrinsic relationships among core variables. Specifically, we controlled for four variables: gender, education, age, and position. The selection of these variables was based on thorough theoretical considerations. First, gender differences may influence the expression of innovative behavior in the workplace through social role expectations (Waqas et al., 2021). Education provides the necessary knowledge foundation and cognitive resources for creative thinking (Lu et al., 2024). Age is often associated with risk-taking propensity and cognitive flexibility, which in turn affect employee creativity (Zhang et al., 2023). Job position directly relates to the degree of work autonomy and resource support employees can access (Caplan, 1987). Therefore, by statistically controlling these potential confounders, we aim to more accurately estimate the net effect of DIHRP on employee creativity through psychological mechanisms, thereby enhancing the internal validity of our findings.

## 4 Results

### 4.1 Common method bias, discriminant validity, and convergent validity

Since the data were all self-assessed by the employees, in order to avoid the effect of common method bias (Podsakoff et al., 2003), the Harman single-factor test found that the unrotated first factor explained 31.096% of the variation, less than 40% (Zhou and George, 2001). Further analysis using the common latent factor method by adding the common method bias factor together with the original five factors showed no significant improvement in the model fit index (△*χ*^2^/df = 0.080, △CFI = 0.005, △TLI = 0.005, △SRMR = 0.007, △RMSEA = 0.004). Therefore, the problem of common method bias is not serious.

In this study, AMOS 25.0 software was used to determine the degree of compliance of the scale question items with the measurement objectives by confirmatory factor analysis. In general, the model is considered to fit well if the *χ*^2^/df is less than 5 (the smaller, the better), the RMSEA is less than 0.1 (the smaller, the better), and the CFI and TLI are all above 0.8 (the larger, the better). According to Table 1, it was found that the five-factor model fit better (*χ*^2^/df = 1.300, CFI = 0.985, TLI = 0.983, SRMR = 0.340, RMSEA = 0.028) than the others, which indicates that the discriminant validity between the core variables in this study was significant.

**Table 1:** The results of confirmatory factor analysis.

Models	*χ*^2^/df	CFI	TFI	SRMR	RMSEA
Five-factor model	1.300	0.985	0.983	0.034	0.028
Four-factor model	4.105	0.841	0.826	0.108	0.091
Three-factor model	6.556	0.713	0.689	0.135	0.122
Two-factor model	9.024	0.583	0.551	0.144	0.147
One-factor model	11.212	0.468	0.429	0.148	0.166

One-factor model: All five variables are loaded onto a single latent factor; Two-factor model: DIHRP, inclusive leadership, supplementary fit, and complementary fit are combined into one factor; Three-factor model: DIHRP, inclusive leadership, and supplementary fit are combined into one factor; Four-factor model: DIHRP and inclusive leadership are combined into one factor; Four-factor model: Each variable is a separate factor.

### 4.2 Descriptive statistics

Descriptive statistics and correlation analysis were shown in Table 2. DIHRP were found to be significantly and positively correlated with employee creativity (*r* = 0.416, *p* < 0.001), supplementary fit (*r* = 0.362, *p* < 0.001), and complementary fit (*r* = 0.390, *p* < 0.001). In addition, supplementary fit showed a significant positive correlation with employee creativity (*r* = 0.560, *p* < 0.001), as did complementary fit (*r* = 0.437, *p* < 0.001). These findings provide preliminary support for Hypotheses H1, H2a, H2b, H3a, and H3b.

**Table 2:** Mean, standard deviation, and correlation coefficient analysis of variables.

Variables	*M*	SD	1	2	3	4	5	6	7	8	9
1. Gender	1.44	0.50	1								
2. Education	2.57	1.05	−0.019	1							
3. Age	2.35	0.86	0.034	−0.319^***^	1						
4. Position	1.51	0.73	0.028	−0.025	−0.009	1					
5. DI-HRP	3.69	0.79	−0.041	0.067	0.009	−0.009	1				
6. Supplementary fit	3.35	0.84	0.075	0.034	0.058	0.011	0.362^***^	1			
7. Complementary fit	3.40	0.83	−0.044	−0.078	0.023	−0.031	0.390^***^	0.430^***^	1		
8. Employee creativity	3.36	0.88	0.054	0.086	0.07	0.006	0.416^***^	0.560^***^	0.437^***^	1	
9. Inclusive leadership	3.43	0.83	−0.031	0.002	0.027	−0.015	0.077	0.204^***^	0.232^***^	0.114^**^	1

*p < 0.05, **p < 0.01, ***p < 0.001.

### 4.3 Hypotheses testing

Direct effect test. The main effect was tested by the hierarchical regression method, and the results were shown in Table 3. The results of Model 2, after controlling for employee gender, age, education, and job position, DIHRP had a significant positive effect on employee creativity (*β* = 0.412, *p* < 0.001), with a significant change in *R*^2^ (△*R*^2^), thereby supporting Hypothesis H1.

**Table 3:** Regression analysis results of the main effect, mediating effect and moderating effect.

Variables	Employee creativity							Supplementary fit			Complementary fit		
	M1	M2	M3	M4	M5	M6	M7	M8	M9	M10	M11	M12	M13
1. Gender	0.052	0.069	0.012	0.073	0.028	0.079	0.076	0.073	0.088	0.097^*^	−0.045	−0.029	−0.019
2. Education	0.121^*^	0.090	0.088	0.157^**^	0.075	0.127^**^	0.08	0.060	0.032	0.022	−0.08	−0.111^*^	−0.121^*^
3. Age	0.107	0.092	0.065	0.107^*^	0.063	0.097^*^	0.098^*^	0.075	0.062	0.065	−0.002	−0.015	−0.014
4. Position	0.009	0.011	0.002	0.023	0.005	0.021	0.002	0.011	0.013	0.007	−0.032	−0.03	−0.036
5. DIHRP		0.412^***^			0.243^***^	0.277^***^	0.431^***^		0.363^***^	0.373^***^		0.396^***^	0.404^***^
6. Supplementary fit			0.552^***^		0.464^***^								
7. Complementary fit				0.451^***^		0.341^***^							
8. Inclusive leadership							0.092^*^			0.187^***^			0.211^***^
DIHRP* Inclusive leadership							0.242^***^			0.222^***^			0.219^***^
*R* ^2^	0.021	0.189	0.322	0.222	0.373	0.286	0.387	0.012	0.143	0.223	0.009	0.165	0.253
△*R*^2^		0.168^***^	0.302^***^	0.201^***^	0.184^***^	0.265^***^	0.376^***^		0.131^***^	0.080^***^		0.156^***^	0.088^***^

*p < 0.05, **p < 0.01, ***p < 0.001.

According to Model 9, DIHRP significantly and positively predicted supplementary fit (*β* = 0.363, *p* < 0.001), with a significant increase in explained variance (△*R*^2^), providing support for Hypothesis H2a.

According to Model 12, DIHRP also showed a significant positive association with complementary fit (*β* = 0.396, *p* < 0.001), and the change in R^2^ was significant, thus supporting Hypothesis H2b.

As shown in Model 3, supplementary fit had a significant positive effect on employee creativity (*β* = 0.552, *p* < 0.001), with a significant △*R*^2^, lending support to Hypothesis H3a.

Similarly, Model 4 demonstrated that complementary fit significantly predicted employee creativity (*β* = 0.451, *p* < 0.001), with a significant change in *R*^2^, thereby supporting Hypothesis H3b.

Mediating effect test. To test the mediating effects, this study employed a three-step hierarchical regression approach. Detailed results are presented in Table 3.

Model 2 examined the direct effect. The results showed that DIHRP had a significant positive effect on employee creativity (*β* = 0.412, *p* < 0.001). Model 9 tested the effect of DIHRP on the proposed mediator. Results indicated that DIHRP significantly predicted supplementary fit (*β* = 0.363, *p* < 0.001). Model 5 included the mediator in the regression predicting employee creativity. The results revealed that supplementary fit had a significant positive effect on employee creativity (*β* = 0.464, *p* < 0.001), while the direct effect of DIHRP on employee creativity decreased (*β* = 0.243, *p* < 0.001). These results indicate that supplementary fit partially mediates the relationship between DIHRP and employee creativity, thereby supporting Hypothesis H4a.

Similarly, for Hypothesis H4b, the first step (Model 2) confirmed the significant effect of DIHRP on employee creativity. Model 12 showed a significant positive effect of DIHRP on complementary fit (*β* = 0.396, *p* < 0.001). Model 6 demonstrated that when complementary fit was included, its effect on employee creativity remained significant (*β* = 0.341, *p* < 0.001), while the coefficient for DIHRP decreased (*β* = 0.277, *p* < 0.001). This provides evidence that complementary fit also partially mediates the relationship between DIHRP and employee creativity, thus supporting Hypothesis H4b.

In addition, we conducted a bootstrapping analysis using Hayes (2013) PROCESS macro (Model 4) to further test the mediating effects. Based on 5,000 resamples, the indirect effect via supplementary fit was 0.1870, and the 95% confidence interval (CI) did not include zero [CI = 0.1180, 0.2566], indicating a significant mediation effect. This provides additional support for Hypothesis H4a. Similarly, the indirect effect via complementary fit was 0.1501, with a 95% confidence interval of [CI = 0.0898, 0.2193], also excluding zero. These results confirm the significance of the mediating role of complementary fit and provide further support for Hypothesis H4b.

Moderating effect test. Before testing the moderation effects of inclusive leadership, all relevant variables were mean-centered. Stepwise regression analysis was then conducted to examine the interaction terms. As shown in Table 3, Model 7 indicates that the interaction between DIHRP and inclusive leadership significantly and positively predicted employee creativity (*β* = 0.242, *p* < 0.001).

**Figure 2:**
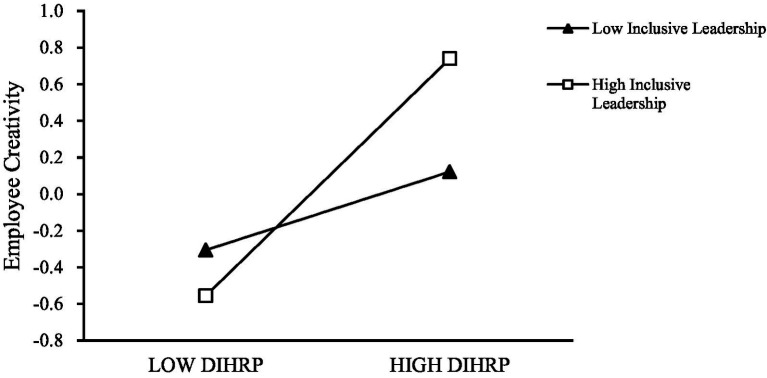
The moderating effect of inclusive leadership between DIHRP and employee creativity.

The simple slope analysis presented in Figure 2 shows that the positive effect of DIHRP on employee creativity was weaker under low levels of inclusive leadership, and stronger under high levels of inclusive leadership. These results confirm the moderating effect of inclusive leadership on the relationship between DIHRP and employee creativity, thereby supporting Hypothesis H5.

Furthermore, Model 10 and Model 13 show that the interaction between DIHRP and inclusive leadership also significantly predicted supplementary fit (*β* = 0.222, *p* < 0.001) and complementary fit (*β* = 0.219, *p* < 0.001), respectively.

**Figure 3:**
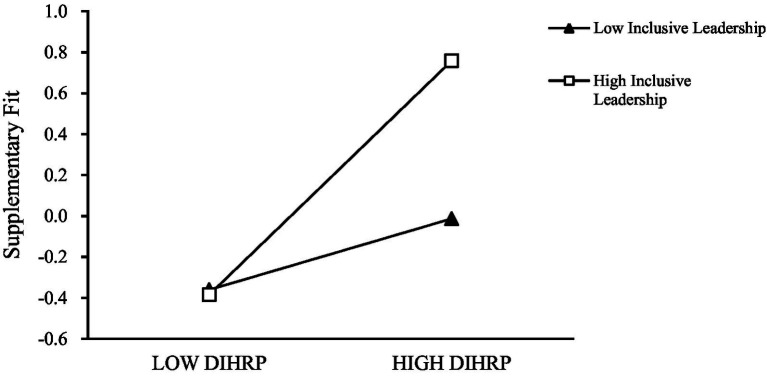
The moderating effect of inclusive leadership between DIHRP and supplementary fit.

**Figure 4:**
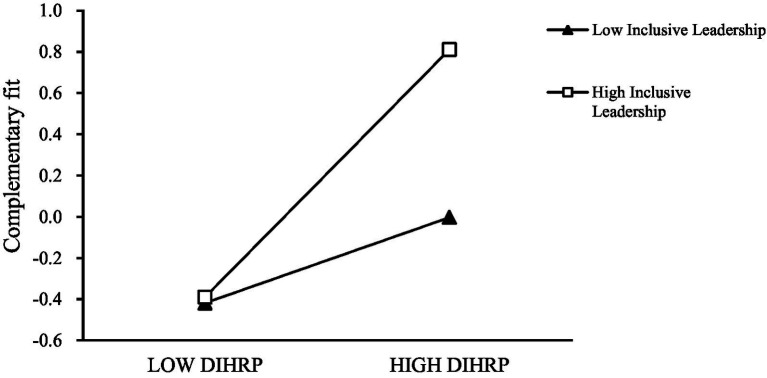
The moderating effect of inclusive leadership between DIHRP and complementary fit.

As illustrated in Figures 3, 4, the simple slope plots demonstrate that the positive effect of DIHRP on supplementary fit is weaker when inclusive leadership is low, and stronger when inclusive leadership is high. Similarly, the effect of DIHRP on complementary fit is also moderated by inclusive leadership, such that the effect is stronger under high levels of inclusive leadership. Together, these findings provide empirical support for Hypotheses H6a and H6b.

To examine the moderated mediation effects, this study employed bootstrapping procedures using the PROCESS macro (Hayes and Rockwood, 2020). Specifically, we tested whether inclusive leadership moderates the indirect effect of DIHRP on employee creativity through supplementary fit and complementary fit. The results are summarized in Table 4.

**Table 4:** Moderated mediation tests.

Mediator variables	Inclusive leadership	Indirect effect	95%CI	
			Low	High
Supplementary fit	Low	0.0896	0.0163	0.1750
	Middle	0.1923	0.1283	0.2616
	High	0.2950	0.1935	0.4005
	Difference	0.1243	0.0453	0.1999
Complementary fit	low	0.0787	0.0180	0.1537
	Middle	0.1532	0.0939	0.2241
	High	0.2277	0.1306	0.3407
	Difference	0.0901	0.0205	0.1693

When supplementary fit served as the mediator, the indirect effect of DIHRP on employee creativity was significantly positive at low, medium, and high levels of inclusive leadership. Moreover, the difference in the indirect effect across leadership levels was significant (95% CI = [0.0453, 0.1999], number of bootstrap samples = 5,000; Effect = 0.1243). These findings provide empirical support for Hypothesis H7a.

Similarly, when complementary fit was examined as the mediator, the indirect effect of DIHRP on employee creativity remained significantly positive across all three levels of inclusive leadership. The index of moderated mediation was statistically significant (95% CI = [0.0205, 0.1693], bootstrap samples = 5,000; Effect = 0.0901), thereby supporting Hypothesis H7b.

## 5 Conclusion

First, this study demonstrates that DIHRP significantly and positively predicts employee creativity. By institutionalizing respect for individual differences, tolerating mistakes, and offering diverse developmental opportunities, DIHRP enhances employees’ sense of self-worth and perceived value realization, which in turn stimulates their creative potential. The workplace is not merely a site of task execution but also a platform for personal meaning-making and identity expression (Andersson and Pearson, 1999). Only when employees feel respected and empowered within the organization can they sustainably engage in creative behavior and generate long-term value for the organization.

Second, both supplementary fit and complementary fit serve as significant mediating mechanisms through which DIHRP fosters employee creativity. Inclusive policies help employees perceive alignment between their personal attributes and the organization’s values and culture, while also fulfilling their developmental needs through competency-building and resource provision. These dual pathways of person–organization fit significantly enhance employees’ creative engagement.

Third, inclusive leadership plays a moderating role in the relationship between DIHRP, employee fit perceptions, and creativity. Under conditions of high inclusive leadership, organizations are more likely to cultivate an atmosphere of openness, trust, and emotional support (Umrani et al., 2023). This amplifies employees’ positive perceptions of formal HR practices and strengthens both supplementary fit and complementary fit, thereby increasing the likelihood of creative behavior.

Fourth, inclusive leadership also significantly enhances the indirect effects of DIHRP on employee creativity via the parallel mediating pathways of supplementary fit and complementary fit. In highly inclusive leadership contexts, DIHRP is more effective in activating employees’ psychological alignment and perceived resource compatibility. These two fit mechanisms jointly function to facilitate the translation of inclusive HR systems into elevated levels of employee creativity.

### 5.1 Theoretical implications

First, this study directly advances the theory of person-organization fit. Although supplementary fit and complementary fit are considered classic dimensions of fit (Kristof, 1996), existing research has mostly explored the impact of overall fit in a general way or focused on only one pathway, lacking a detailed deconstruction of how the two serve as parallel mediating mechanisms within the same framework. The dual-pathway model of “supplementary-complementary” constructed in this study reveals that DIHRP can influence employee creativity through both pathways. This not only responds to the call for “specification” of fit dimensions in fit theory (Cable and DeRue, 2002) but also, by deeply integrating SIT and COR theory with supplementary and complementary fit, respectively, it provides an integrated theoretical framework for understanding the underlying dynamics of the different fit dimensions (Raza and Khan, 2022). We clearly demonstrate that supplementary fit primarily satisfies individuals’ needs for belonging and identity, while complementary fit primarily satisfies their needs for resources and growth. Together, these two constitute the comprehensive psychological foundation for individuals to gain support from their organizational environment and, in turn, stimulate employee creativity. Therefore, by revealing the synergistic mediating mechanisms of dual fit, it enriches and deepens our theoretical understanding of how person-organization fit is activated and operates.

Second, this study provides new insights into the strategic human resource management literature. While previous research has focused on green HRP and work-family balance HRP (Hu et al., 2025), this study identifies diversity and inclusion as a distinct strategic orientation and systematically explores the mechanisms underlying this emerging construct. By tracing its mechanisms back to the dual pathway of person-organization fit, we reveal a unique value proposition of DIHRP that distinguishes it from other HR systems: it integrates employees by fostering shared inclusive values (the supplementary fit) and empowers them by providing differentiated resources and opportunities (the complementary fit). This approach defies previous research’s overemphasis on “best practices” or “universal” HR practices and emphasizes that HR systems based on specific strategic values (such as inclusion) drive employee behavior by activating corresponding psychological mechanisms, thus enriching our understanding of the “heterogeneity effect” of strategic HR management systems.

Third, this study deepens our understanding of the synergistic effects of human resource systems and leadership by identifying the moderating role of inclusive leadership. The study found that inclusive leadership not only strengthens the direct effect of DIHRP on creativity but also specifically enhances the path from perceived fit to creativity. This finding suggests that formal institutional systems and informal leadership support complement and reinforce each other in influencing employee psychology and behavior: DIHRP creates a fair institutional environment and resource platform, while inclusive leadership, through its daily, interactive behavior, provides critical psychological safety for employees to internalize institutional signals and engage in creative behaviors based on perceived fit. This enriches human resource management process theory from the perspective of “institution-leadership” interaction and adds an important boundary condition to person-organization fit theory: whether the perception of fit translates into positive extra-role behavior depends on the micro-context fostered by the direct leader (Park et al., 2019).

### 5.2 Practical implications

First, organizations should establish an institutionalized and operational DIHRP. In the context of global economic integration, driving the increasing diversity of the workforce, organizations urgently need to tap individual potential and stimulate creativity through structured, inclusive practices. Specifically, organizations should formulate clear, measurable, and enforceable human resource policies with respect for differences, equal opportunities, and tolerance as core principles. These policies should cover the entire process of recruitment, training, performance evaluation, and rewards, aiming to cultivate a psychologically safe and participatory organizational atmosphere. When implementing in the Chinese context, organizations should fully consider the characteristics of a high power distance culture and adopt a strategy of “combining top-level design with cultural immersion”: on the one hand, by incorporating diversity and inclusion indicators into the manager assessment system, ensure policy implementation; on the other hand, through flexible methods such as setting inclusive models and telling success stories, integrate the concepts of diversity and inclusion into the organizational culture, achieve a transformation from formal acceptance to inner recognition, and thus stimulate employee creativity.

Second, organizations should systematically strengthen employee-organization supplementary fit and complementary fit. This research finds that these two forms of person-organization fit are key mechanisms linking DIHRP and employee creativity, providing actionable insights for talent management strategies. In practice, organizations can enhance alignment by implementing values-based selection processes during recruitment; integrating organizational values and behavioral norms into training and development; and designing evaluation mechanisms that encourage experimentation and embrace failure within performance management and incentive systems. Given the Chinese employee’s emphasis on collective belonging and career stability, organizations should prioritize integrating the traditional wisdom of “harmony in diversity” into their values development. They should also strengthen employee-organization fit in terms of resource needs through long-term development systems, such as mentoring and apprenticeship programs, and clear internal promotion pathways.

Third, organizations should leverage inclusive leadership as an emotional bridge between institutional practices and employee experience. This research shows that inclusive leadership not only strengthens the impact of inclusive HR practices on person-organization fit and creativity but also plays a key role in providing emotional support and building a shared identity. In practice, organizations should incorporate inclusive leadership development into leadership training programs to enhance managers’ emotional intelligence, communication skills, and relationship awareness. In light of China’s relationship-oriented culture, we recommend focusing on cultivating managers’ leadership wisdom of “ren” (empathy and care) and “li” (respect for propriety). Through localized communication methods such as “heart-to-heart talks,” managers can proactively understand employee needs, foster a psychologically safe innovation environment while maintaining organizational authority, and truly implement inclusive policies within an organizational environment that prioritizes “face” and “harmony.”

### 5.3 Limitations and future research

First, the data relied primarily on self-reported measures, which may raise concerns about common method bias (CMB). Key constructs—such as perceptions of inclusive HR practices, person–organization fit, and creativity—were assessed based on employees’ subjective evaluations. Although statistical controls were applied to test and mitigate CMB, the potential for inflated associations due to shared method variance cannot be entirely ruled out. Future research is encouraged to incorporate multi-source data—such as supervisor ratings, archival HR documents, or objective performance indicators—to enhance measurement validity and robustness.

Second, the sample was drawn primarily from Chinese organizations, which may limit the external validity of the findings across cultural contexts. The implementation and interpretation of both inclusive HR practices and inclusive leadership in China may be shaped by Confucian values, relational orientations, and high-power distance norms. In other cultural or institutional settings, employees may differ in how they perceive “inclusion” and “fit” and how these perceptions translate into creative behavior. Future research should therefore consider cross-cultural comparative studies to explore the cultural adaptability and contextual transferability of inclusive HR systems, thereby strengthening the cross-cultural generalizability of the findings.

Third, the current study focuses primarily on the direct and indirect effects of inclusive HR practices on employee creativity. However, there remains room to expand the model structure. Future research may incorporate individual-level traits (e.g., psychological safety, intrinsic motivation) or team-level constructs (e.g., team diversity, inclusive team climate) as potential mediators or moderators. This would enable the construction of more complex, multilevel models to better capture the cross-level mechanisms through which inclusive HR systems influence creativity across individual, team, and organizational levels.

## Data Availability

The raw data supporting the conclusions of this article will be made available by the authors, without undue reservation.

## References

[ref1] Ahmed F. Hu W. Arslan A. Huang H. (2024). Ambidexterity of HR practices in fortune 500 companies and employee innovation performance: mediating role of inclusive leadership. J. Organ. Chang. Manage 37, 237–254. doi: 10.1108/JOCM-05-2022-0139

[ref2] Andersson L. M. Pearson C. M. (1999). Tit for tat? The spiraling effect of incivility in the workplace. Acad. Manag. Rev 24:452. doi: 10.2307/259136

[ref3] Ashforth B. E. Mael F. (1989). Social identity theory and the organization. Acad. Manag. Rev. 14, 20–39. doi: 10.2307/258189

[ref4] Aust I. Matthews B. Muller-Camen M. (2020). Common good HRM: a paradigm shift in sustainable HRM? Hum. Resour. Manag. Rev. 30, 100–105. doi: 10.1016/j.hrmr.2019.100705

[ref5] Baer M. Oldham G. R. (2006). The curvilinear relation between experienced creative time pressure and creativity: moderating effects of openness to experience and support for creativity. J. Appl. Psychol. 91, 963–970. doi: 10.1037/0021-9010.91.4.963, PMID: 16834519

[ref6] Brislin R. W. (1970). Back-translation for cross-cultural research. J. Cross-Cult. Psychol. 1, 185–216. doi: 10.1177/135910457000100301

[ref7] Brown R. (2000). Social identity theory: past achievements, current problems and future challenges. Eur. J. Soc. Psychol. 30, 745–778. doi: 10.1002/1099-0992

[ref8] Burmeister A. van der Heijden B. Yang J. Deller J. (2018). Knowledge transfer in age-diverse coworker dyads in China and Germany: how and when do age-inclusive human resource practices have an effect? Hum. Resour. Manag. J. 28, 605–620. doi: 10.1111/1748-8583.12207

[ref9] Cable D. M. DeRue D. S. (2002). The convergent and discriminant validity of subjective fit perceptions. J. Appl. Psychol. 87, 875–884. doi: 10.1037/0021-9010.87.5.875, PMID: 12395812

[ref10] Caplan R. D. (1987). Person-environment fit theory and organizations: commensurate dimensions, time perspectives, and mechanisms. J. Vocat. Behav. 31, 248–267. doi: 10.1016/0001-8791(87)90042-X

[ref11] Carmeli A. Reiter-Palmon R. Ziv E. (2010). Inclusive leadership and employee involvement in creative tasks in the workplace: the mediating role of psychological safety. Creativ. Res. J. 22, 250–260. doi: 10.1080/10400419.2010.504654

[ref12] Farmer S. M. Tierney P. Kung-McIntyre K. (2003). Employee creativity in Taiwan: an application of role identity theory. Acad. Manag. J. 46, 618–630. doi: 10.2307/30040653

[ref13] Gümüsay A. A. Smets M. Morris T. (2020). “God at work”: engaging central and incompatible institutional logics through elastic hybridity. Acad. Manag. J. 63, 124–154. doi: 10.5465/amj.2016.0481

[ref14] Halbesleben J. R. Wheeler A. R. (2015). To invest or not? The role of coworker support and trust in daily reciprocal gain spirals of helping behavior. J. Manag. 41, 1628–1650. doi: 10.1177/0149206312455246

[ref15] Hayes A. F. (2013). Introduction to mediation, moderation, and conditional process analysis: a regression-based approach. New York, NY: Guilford Press.

[ref16] Hayes A. F. Rockwood N. J. (2020). Conditional process analysis: concepts, computation, and advances in the modeling of the contingencies of mechanisms. Am. Behav. Sci. 64, 19–54. doi: 10.1177/0002764219859633

[ref17] Hobfoll S. E. (1989). Conservation of resources: a new attempt at conceptualizing stress. Am. Psychol. 44, 513–524, PMID: 2648906 10.1037//0003-066x.44.3.513

[ref18] Hu W. Ahmed F. Su Y. (2023). Transactive memory system and entrepreneurial team performance: the impact of ability to improvise and market competition. Int. J. Emerg. Mark. 18, 6234–6259. doi: 10.1108/IJOEM-09-2021-1340

[ref19] Hu W. Xu Y. Yang J. (2025). Green human resource practices and corporate sustainable performance—the role of corporate green culture and dynamic capabilities. Corp. Soc. Responsib. Environ. Manag. 32, 635–646. doi: 10.1002/csr.2978

[ref21] Jia N. Luo X. Fang Z. Liao C. (2024). When and how artificial intelligence augments employee creativity. Acad. Manag. J. 67, 5–32. doi: 10.5465/amj.2022.0426

[ref22] Kahn W. A. (1990). Psychological conditions of personal engagement and disengagement at work. Acad. Manag. J. 33, 692–724. doi: 10.2307/256287

[ref23] Kristof A. L. (1996). Person-organization fit an integrative review of its conceptualizations, measurement, and implications. Pers. Psychol. 49, 1–49. doi: 10.1111/j.1744-6570.1996.tb01790.x

[ref24] Lu S. Sun Z. Huang M. (2024). The impact of digital literacy on farmers’ pro-environmental behavior: an analysis with the theory of planned behavior. Front. Sustain. Food Syst. 8:1432184. doi: 10.3389/fsufs.2024.1432184

[ref25] Moore K. McDonald P. Bartlett J. (2017). The social legitimacy of disability inclusive human resource practices: the case of a large retail organisation. Hum. Resour. Manag. J. 27, 514–529. doi: 10.1111/1748-8583.12129

[ref26] Muchinsky P. M. Monahan C. J. (1987). What is person-environment congruence? Supplementary versus complementary models of fit. J. Vocat. Behav. 31, 268–277. doi: 10.1016/0001-8791(87)90043-1

[ref27] Orekoya I. O. (2023). Inclusive leadership and team climate: the role of team power distance and trust in leadership. Leadersh. Org. Dev. J. 45, 94–115. doi: 10.1108/LODJ-03-2023-0142

[ref28] Park O. Bae J. Hong W. (2019). High-commitment HRM system, HR capability, and ambidextrous technological innovation. Int. J. Hum. Resour. Manag. 30, 1526–1548. doi: 10.1080/09585192.2017.1296880

[ref29] Pless N. Maak T. (2004). Building an inclusive diversity culture: principles, processes and practice. J. Bus. Ethics 54, 129–147. doi: 10.1007/s10551-004-9465-8

[ref30] Podsakoff P. M. Mackenzie S. B. Lee J.-Y. Podsakoff N. P. (2003). Common method biases in behavioral research: a critical review of the literature and recommended remedies. J. Appl. Psychol. 88, 879–903. doi: 10.1037/0021-9010.88.5.879, PMID: 14516251

[ref31] Raza S. A. Khan K. A. (2022). Impact of green human resource practices on hotel environmental performance: the moderating effect of environmental knowledge and individual green values. Int. J. Contemp. Hosp. Manag. 34, 2154–2175. doi: 10.1108/IJCHM-05-2021-0553

[ref32] Roberson Q. Perry J. L. (2022). Inclusive leadership in thought and action: a thematic analysis. Group Organ. Manage. 47, 755–778. doi: 10.1177/10596011211013161

[ref33] Rudolph C. W. Zacher H. (2021). Age inclusive human resource practices, age diversity climate, and work ability: exploring between- and within-person indirect effects. Work Aging Retire. 7, 387–403. doi: 10.1093/workar/waaa008

[ref34] Silva P. Moreira A. C. Mota J. (2022). Employees’ perception of corporate social responsibility and performance: the mediating roles of job satisfaction, organizational commitment and organizational trust. J. Strategy. Manag. 16, 92–111. doi: 10.1108/JSMA-10-2021-0213

[ref35] Umrani W. A. Bachkirov A. A. Nawaz A. Ahmed U. Pahi M. H. (2023). Inclusive leadership, employee performance and well-being: an empirical study. Leadersh. Org. Dev. J. 45, 231–250. doi: 10.1108/LODJ-03-2023-0159

[ref36] Wang L. Zhang Q. Wong P. P. W. (2024). Reexamination of consumers’ willingness to stay at green hotels: rethinking the role of social identity theory, value-belief-norm theory, and theory of planned behavior. J. Hosp. Mark. Manag. 33, 547–581. doi: 10.1080/19368623.2023.2292639

[ref37] Waqas M. Yahya F. Ahmed A. Rasool Y. Hongbo L. (2021). Unlocking employee’s green behavior in fertilizer industry: the role of green HRM practices and psychological ownership. Int. Food Agribus. Manag. Rev. 24, 827–844. doi: 10.22434/IFAMR2020.0109

[ref38] Wightman G. B. Christensen R. K. (2025). A systematic review of person-environment fit in the public sector: theorizing a multidimensional model. Public Adm. Rev. 85, 386–401. doi: 10.1111/puar.13843

[ref39] Wray E. Sharma U. Subban P. (2022). Factors influencing teacher self-efficacy for inclusive education: a systematic literature review. Teach. Teach. Educ. 117:103800. doi: 10.1016/j.tate.2022.103800

[ref40] Zhang M. Shi H. Williams L. Lighterness P. Li M. Khan A. U. (2023). An empirical test of the influence of rural leadership on the willingness to participate in public affairs from the perspective of leadership identification. Agri 13:1976. doi: 10.3390/agriculture13101976

[ref41] Zhao F. Q. Chen Y. Xiang H. D. (2022). Research on the impact of diverse and inclusive human resource practices on employees’ innovative behaviors: the role of work prosperity and shared leadership. Sci. Res. Manag. 43, 192–200. doi: 10.19571/j.cnki.1000-2995.2022.08.022

[ref42] Zhao F. Hu W. Ahmed F. Huang H. (2023). Impact of ambidextrous human resource practices on employee innovation performance: the roles of inclusive leadership and psychological safety. Eur. J. Innov. Manag. 26, 1444–1470. doi: 10.1108/EJIM-04-2021-0226

[ref43] Zhao F. Q. Lu Q. Chen Y. (2020). A research on the impact of diverse inclusive human resource practice on individual creativity—the effect of ambidextrous learning and charismatic leadership. Sci. Res. Manag. 41, 94–102. doi: 10.19571/j.cnki.1000-2995.2020.04.010

[ref44] Zhou J. George J. M. (2001). When job dissatisfaction leads to creativity: encouraging the expression of voice. Acad. Manag. J. 44, 682–696. doi: 10.5465/3069410

